# Emerging Applications of Nanobiosensors in Pathogen Detection in Water and Food

**DOI:** 10.3390/bios13100922

**Published:** 2023-10-11

**Authors:** Hiram Martin Valenzuela-Amaro, Alberto Aguayo-Acosta, Edgar Ricardo Meléndez-Sánchez, Orlando de la Rosa, Perla Guadalupe Vázquez-Ortega, Mariel Araceli Oyervides-Muñoz, Juan Eduardo Sosa-Hernández, Roberto Parra-Saldívar

**Affiliations:** 1 Tecnologico de Monterrey, Institute of Advanced Materials for Sustainable Manufacturing, Monterrey 64849, Mexico; amaro.hiram@tec.mx (H.M.V.-A.); aguayo.alberto@tec.mx (A.A.-A.); edgar.rmelendez@tec.mx (E.R.M.-S.); orlando.delarosa@tec.mx (O.d.l.R.); mariel.oyervides@tec.mx (M.A.O.-M.); 2 Tecnologico de Monterrey, School of Engineering and Sciences, Monterrey 64849, Mexico; 3 Tecnologico Nacional de México, Instituto Tecnológico de Durango, Durango 34080, Mexico; pvazquez@itdurango.edu.mx

**Keywords:** nanobiosensors, nanomaterials, foodborne diseases, waterborne diseases, food safety

## Abstract

Food and waterborne illnesses are still a major concern in health and food safety areas. Every year, almost 0.42 million and 2.2 million deaths related to food and waterborne illness are reported worldwide, respectively. In foodborne pathogens, bacteria such as *Salmonella*, Shiga-toxin producer *Escherichia coli*, *Campylobacter*, and *Listeria monocytogenes* are considered to be high-concern pathogens. High-concern waterborne pathogens are *Vibrio cholerae*, leptospirosis, *Schistosoma mansoni,* and *Schistosima japonicum*, among others. Despite the major efforts of food and water quality control to monitor the presence of these pathogens of concern in these kinds of sources, foodborne and waterborne illness occurrence is still high globally. For these reasons, the development of novel and faster pathogen-detection methods applicable to real-time surveillance strategies are required. Methods based on biosensor devices have emerged as novel tools for faster detection of food and water pathogens, in contrast to traditional methods that are usually time-consuming and are unsuitable for large-scale monitoring. Biosensor devices can be summarized as devices that use biochemical reactions with a biorecognition section (isolated enzymes, antibodies, tissues, genetic materials, or aptamers) to detect pathogens. In most cases, biosensors are based on the correlation of electrical, thermal, or optical signals in the presence of pathogen biomarkers. The application of nano and molecular technologies allows the identification of pathogens in a faster and high-sensibility manner, at extremely low-pathogen concentrations. In fact, the integration of gold, silver, iron, and magnetic nanoparticles (NP) in biosensors has demonstrated an improvement in their detection functionality. The present review summarizes the principal application of nanomaterials and biosensor-based devices for the detection of pathogens in food and water samples. Additionally, it highlights the improvement of biosensor devices through nanomaterials. Nanomaterials offer unique advantages for pathogen detection. The nanoscale and high specific surface area allows for more effective interaction with pathogenic agents, enhancing the sensitivity and selectivity of the biosensors. Finally, biosensors’ capability to functionalize with specific molecules such as antibodies or nucleic acids facilitates the specific detection of the target pathogens.

## 1. Introduction

Every year, contaminated food is responsible for 420,000 deaths and 600 million cases of foodborne illnesses caused by spoiled food [1]. This is not just a problem in low–middle-income countries, high-income countries also have several troubles related to foodborne pathogens. In the U.S. alone, there are more than 9.4 million deaths per year due to the ingestion of pathogenic bacteria in food [2]. During 2010, 420,000 people (one-third of them being children under the age of five) died from illnesses related to salmonellosis and *Escherichia coli* infections [3]. Foodborne illnesses arise from the presence of pathogens, toxins, or contaminants in food products, and are typically associated with gastrointestinal symptoms (diarrhea, vomiting, abdominal pain, and fever), and other adverse effects on human health such as neurological, hepatic, and renal complications, even becoming a life-threatening issue if not appropriately addressed [4,5]. In recent years, the majority of reported foodborne illness outbreaks were caused by pathogens such as Norovirus [5], *Campylobacter* [6], *Salmonella* [5,6], *Listeria monocytogenes* [7], and Shiga toxin-producing *E. coli* [8]. Less frequently reported but still of concern are the pathogens *Staphylococcus aureus* [9], *Clostridium* species [10], *Bacillus cereus* [11], and *Yersinia enterocolitica* [12].

Similarly to food safety, the presence of pathogens in water is a major issue for public health [13]. It is estimated that 663 million people consume unsafe water from surface or groundwater sources [14]. More than 2.2 million deaths per year and more cases of illness (diarrhea, gastrointestinal, and systematic diseases) are linked to contaminated water ingestion [15]; the pathogens of greatest concern are *Salmonella*, *Shigella*, *Campylobacter*, *S. aureus*, and *E. coli* [16,17]. However, viruses and parasites are becoming a problem for water security [18]. Parasites and viruses linked to waterborne outbreaks include *Vibrio cholerae*, *Leptospira*, *Schistosoma mansoni*, and *Schistosoma japonicum* [16,19,20].

Monitoring the presence of pathogens in water is particularly important as a disease-preventive measure from waterborne illnesses and to monitor water quality. This can be achieved through applying wastewater-based surveillance protocols, which allow the detection of pathogens using molecular biology tools [21,22], which can be applied to verify the discharged water quality and indicate the treatment required to prevent adverse effects on the environment; ensuring water sustainability for future generations.

Pathogen-detection methods play a crucial role in ensuring food and water safety; however, actual monitoring methods are time-consuming processes that usually take days to obtain a precise result [23], making them ineffective for real-time monitoring [24]. In fact, the identification of pathogens such as bacteria and viruses is carried out by gold-standard methodologies, which are traditional techniques such as viable plate counts, flow cytometry, and staining methods, among others [25,26,27]. Nevertheless, the detection time is one of the major limitations of this technique because these techniques require the growth of the microorganism in laboratory conditions (this has not been a limitation per se), which can take several days to produce a result, hindering the response time for the control of pathogens [26]. Techniques based on molecular biology that are used for pathogen detection involve [28] polymerase chain reaction techniques (PCR) [21,29,30,31], multiplex polymerase chain reaction (mPCR) [32], quantitative polymerase chain reaction (qPCR) [33], digital droplet PCR (ddPCR) [34], fluorescence in situ hybridization (FISH) [31], enzyme-linked immunosorbent assay (ELISA) [35], surface-enhanced Raman spectroscopy (SERS) [36], immunological methods [37], next-generation sequencing [38], whole-genome sequencing [39], flow cytometry [40], and surface plasmon resonance imaging (SPR) [41]; these techniques have already been applied as detection methodologies of pathogens in food and water matrices [5,26].

Despite the application of molecular-biology techniques in food and water security, if we consider the technological development of the health sector related to pathogen detection, this sector has already developed advanced technologies such as biosensors with nanomaterials and the incorporation of informatic technologies [42]. Efforts are being conducted in the hope of bringing about more specific and faster methodologies to produce a rapid-response diagnosis and prevent outbreaks, focusing on nanomaterials such as glyconanomaterials [43], nanoparticles [44], ZnO nanorods, nanoconjugate (Au–Fe_3_O_4_), silicon nanonet FET, nanosphere (RNs@Au) in a biosensor device, combined with molecular detection methods (ELISA, qPCR) and also incorporated with informatic technologies, which are used to create more-sensible and appropriate in situ detection systems for pathogens of major concern. This technology has been applied in order to improve the health care system’s response to pathogen-presence emergencies, (as reviewed by Jian et al., 2021) [45] for HIV and Influenza A virus. These technologies have also been applied to Ebola [46], Malaria [47], Dengue virus [43], and in recent years in SARS-CoV-2 monitoring protocols [42]. Considering the advances made in health security and the demands for improved food and water safety, these existing technologies in the health sector should be transferred to other sectors such as food and water security.

For the above mentioned, and the increase in pathogens related to food and water-borne illnesses, the development of pathogen-detection methods is becoming an urgent step to ensuring health and safety [48]. Unfortunately, and despite recent advances in new pathogen-detection approaches, the application of nanomaterials and biosensors is still limited, this is why technologies capable of obtaining better results, in a fast and affordable way, have been studied, resulting in novel technologies, such as biosensor devices, with “rapid, sensitive and specific” protocol for pathogen detection, resolving the priority assignment of ensuring health security, preventing food- and water-ingestion-related outbreaks [49], with even more affordable technology with the inclusion of the use of biosensors and NPs in recent years [44,50,51].

The previously mentioned methods help to perform faster monitoring (real-time surveillance systems) [52], reducing response times of pathogen detection in water [53]. Additionally, the use of biosensors improved with NPs enhanced the detection performance of the device making it a faster, more specific, and portable device [54]. In fact, due to the diversity of the detection capabilities of nanoparticles, they are the subject of many studies that attempt to understand their role when incorporated into pathogen-detection systems [55].

The basic components of a biosensor device are a biorecognition element, a transducer, an amplifier, and a processor component. The biorecognition element recognizes the analyte of interest, the transducer generates a signal from the recognition of the biomarker into a measurable signal, then the signal is processed using the processor and amplifier component, to obtain a signal output [56,57]. In summary, it is a bioanalytical device that detects specific biomarkers using biochemical reactions [58], mediated by isolated enzymes, antibodies, tissues, organelles, or whole cells for pathogen detection, using electrical, thermal, or optical signals [59], which are able to correlate the presence of specific pathogen and signal emission measures [50].

As is already mentioned, the biosensor application has garnered attention in the field of pathogen detection due to their attractive characteristics, such as precision, selectivity and fast analysis [27]. Nevertheless, it is necessary to mention that these methodologies have certain disadvantages, such as the use of expensive enzymes and equipment, including the extensive workflow required for the device’s development. However, these technologies have a promising future due to their potential application in pathogen-rapid-detection methods [53]. Currently, biosensor-based technology has proved its worth due to its unique sensitivity, low detection limit, and simple operation [60].

In the last decade, the biosensors’ structure has been focused on the miniaturization of the devices without affecting the detection efficacy. To achieve this, NPs have been included in the biosensor architecture, resulting in the development of a nanoscale platform. Indeed, in the different sections of the biosensor, NPs are used as signal transducers to convert a biomolecular interaction into an electrical, optical, or magnetic signal [61]. This functionality inside the biosensor is because of unique properties at the nanometric scales (surface area, small size, affinity for some biomolecules, catalytic activity, and autofluorescence) [62,63].

**Figure 1:**
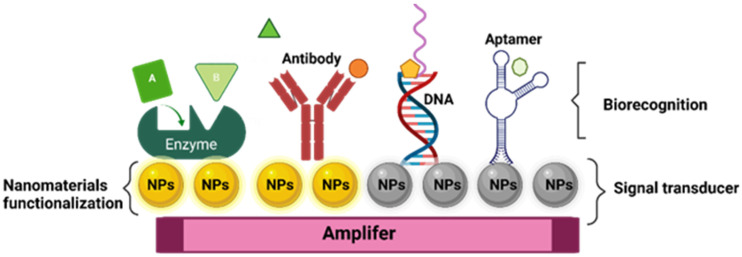
General structure of nanobiosensor with different agents of biorecognition.

Like traditional biosensor devices, the nanobiosensors are composed of three main sections: a biorecognition probe, transducer, and amplifier [64] (Figure 1). The NPs are often in the transducer’s component, helping to enhance the biochemical, electrical, magnetic, or optical signal transduction [61]. Also, these signals can be read simply and effectively as a result of the incorporation of functionalized NPs into the biorecognition component [65].

In fact, nanomaterials have been identified as candidates to enhance biosensors’ sensitivity, improving the detection limits and increasing detection specificity [54,55]. The foregoing is based on the fact that the specificity of signal recognition results in the adequate selection of functionalized ligands with NPs, improving the biomarker attraction; also, NPs convert signals from one form to another or act as detectors of the generated signals [66,67]. Biosensors have several methodologies to acquire relevant signals; for example, the electrochemical biosensors work under the method of capitalizing on reactions between immobilized biomolecules and the biomarker, resulting in electron/ion generation/consumption, modifying the electrical properties of the solution, and resulting in a measurable electrical current [68]. On the other hand, optical biosensors work under the method of discerning variations in light properties (absorption, transmission, and reflection), triggered by physical or chemical interactions with biorecognition elements. These biosensors are categorized into two major groups: label-free, where signals arise directly from analyte interactions, and label-based, employing techniques such as calorimetry, fluorescence, or luminescence to produce detectable optical signals. Both methodologies are available to be applied in diverse areas for pathogen detection [69,70,71].

Other possible classifications of biosensors are based on the type of biorecognition immobilized on the nanomaterial [72], which is divided into the following: enzymes [73], antibodies [74], antigens [75], DNA-RNA [76], organelle [77], cell membrane [78], and phage particle [79]. The conversion of this signal can be achieved using different methods, and this can be classified according to the type of conversion used [80]. Finally, the signal conversion section can include the following optical systems: [69] electrochemical nanobiosensors [81], thermoelectric [82], and piezoelectric [83].

## 2. Biofunctionalization of Nanostructured Surfaces for Interaction with Biorecognition Agents

In order to allow protein adsorption without altering the natural structure of the bioactive molecule, it is essential for the biomaterial surface to be biocompatible [84]. To ensure this, a bioconjugation protocol is applied in the biosensor. Bioconjugation involves the interaction of chemical or functional groups between NPs and biomolecules [85,86]. Additionally, it is important to mention that the properties can affect the efficiency of the connection with the biomarkers, including the optimal distance between biorecognition molecules and the nanostructure, the pH of the storage buffer used, as well as potential modifications to the biological and antigenic properties of biomolecules after conjugation. Hence, it is relevant to develop different approaches to nanostructure conjugation [87,88], using custom functional groups (such as primary amines, carboxylates, cis-diols, and sulfhydryls) on nanosurfaces [62,89].

**Figure 2:**
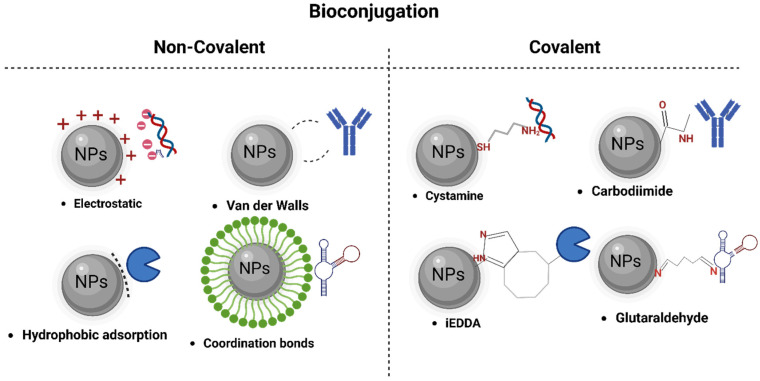
Different techniques of bioconjugation in nanomaterials.

Therefore, the adsorption methods of bioactive molecules into biosensor and nanobiosensor devices can be classified into the following categories: (i) Non-covalent immobilization strategies, based on electrostatic interaction, hydrophobic adsorption, and coordination bond formation between biomarker and the surface of nanomaterials (Figure 2) [86,90]. (ii) Covalent immobilization strategies, involving chemical bonds, chemically activate and modify biorecognition molecules, achieving a stable binding. They enhance sensitivity and selectivity in pathogen detection, ensuring robust interaction [91]. Finally, (iii) a combination of the above-mentioned techniques [87,92].

### 2.1. Biorecognition Section of Bionsensor Device: Enzymes Applications

Enzymatic reactions in the biodetection processes involve the following steps: Firstly, enzymes recognize and bind to the target molecule in the environment/solution, through specific binding sites or active sites on the enzyme. Enzymes are typically immobilized on the surface, electrode, or substrate of the sensor to provide ideal conditions to react with a target molecule and produce a detectable signal [93]. Subsequently, enzymatic activity can be used as a signal through variations in the concentration of protons, entrance or exit of gases, and heat emission [94]. Finally, the signal generated is detected and quantified using a biosensor-detection component (electrochemical and fluorescence techniques) [93,94]. In fact, a biosensor base in enzymes immobilized on Au-NP was used to detect *Campylobacter jejuni* in chicken breast samples; in this biosensor, the nuclease, enzymes, and deoxyribozymes were immobilized to detect the pathogen for the reaction of the scission–enzyme, generating a heteroduplex of DNA–RNA, which finally induced a detectable signal based in a fluorescence-detection model with a limit of detection (LOD) of 10 pM. The viability of this method of DNA detection is assessed as an ultra-sensitive analysis; also, the authors remark that the Au-NP-based detection method can reach the lowest LOD (1 pM) of DNA in samples, one fold less than that required by the already mentioned ultra-sensitive fluorescence-detection method [95]. Also, a comparative analysis of the Influenza A virus detection using a biosensor-based technique showed an LOD of 1 pM mL^−1^ [96], which is extremely low compared to the concentration required in qPCR detection methods [97].

To enhance the biosensor-recognition capacities, the immobilization and stabilization of the enzymes are normal processes. The enzyme immobilization techniques commonly applied to nanostructures are covalent binding, crosslinking, and self-assembled monolayers [98]. However, using enzymes as biorecognition agents has certain disadvantages for in situ applications. Enzymatic activity can be influenced by environmental conditions such as temperature and pH, which affect the stability of the biosensor [99].

### 2.2. Antibody Applications

Antibodies (immunoglobulins) are proteins produced by cells of the immune system called B lymphocytes [100]. They consist of a basic structure composed of four polypeptide chains: two identical heavy chains and two identical light chains with their typical “Y” shape [100,101]. Antibodies are biological molecules, derived from animals, that have gained importance in pathogen-biomarker detection methods, due to their high specificity and in vivo uniqueness [102], designing monoclonal antibodies to precisely target antigens or receptors [103]. An antibody-based biosensor was presented by Majid et al. (2019) [104]. In this type of biosensor, the immobilization of antibodies in gold-NP or nanomaterial are related to weak electrostatic, hydrophobic, or van de Waals force interactions [105]. On the other hand, the preferred method for immobilizing antibodies on NPs and other surfaces is through covalent bonding, specifically using carbodiimide chemistry and maleimide conjugation. This approach allows for longer-lasting and reusable devices, as well as better control over antibody orientation, resulting in enhanced detection capabilities [105,106].

This type of antibody-functionalized biosensor has been used for different pathogen detection, as reported by Guo et al. (2020) [107], who developed a method using NP etching. These techniques allow for the specific detection of *Salmonella Typhimurium*, using catalase-modified antibodies that bind to the bacteria and catalyze the conversion of H_2_O_2_ to H_2_O. In the absence of *S. Typhimurium*, the catalase-modified antibodies do not bind to the bacteria, resulting in a significant accumulation of residual H_2_O_2_. Horseradish peroxidase (HRP) triggers the production of •OH, causing a color change in the Au nanowires from dark blue to pink. The linear detection range is between 18 CFU mL^−1^ and 1.8 × 10^5^ CFU mL^−1^, with a detection limit of 35 CFU mL^−1^ [107]. However, antibody-functionalized biosensors have limitations including lack of specificity, long-term stability, high production cost, challenges in antibody immobilization, potential cross-reactivity, limited antibody availability, and batch-to-batch variability [50].

### 2.3. DNA Applications

Nucleic acid-based biosensors, such as DNA, stand out as biorecognition elements due to their simplicity, speed, and high specificity. For this reason, they are widely used for the detection of pathogens and other substances of interest in various biodetection applications [88]. These characteristics make DNA a powerful and versatile tool in the field of biodetection [108,109]. These molecular probes can be used in different ways in methods such as DNAzyme [110], DNA hairpin [111], DNA hybridization [112], and DNA origami [113]. It is widely recognized that DNA and its assembly structure can be applied to detect specific targets, including nucleic acids, proteins, metal ions, and small biological molecules. Common bioreceptors in this category include deoxyribonucleic acid (DNA), ribonucleic acid (RNA), and peptide nucleic acids (PNA) [114].

These biomarkers have been functionalized with nanomaterials to enhance their selectivity and durability in pathogen biodetection. An application of pathogenic bacteria detection in milk was developed using a combination of photo-induced electron transfer (PET) between a G-quadruplex DNAzyme and silver nanocluster-labeled DNA, along with exponential circular amplification based on the hairpin probe, achieving an ultra-low detection limit of 8 CFU mL^−1^ for *S. Typhimurium*. This strategy represents a promising platform for highly sensitive and specific detection of pathogenic bacteria in food analysis [115]. In another study, a fluorescent DNA hairpin template was developed by designing two hairpin probes with Au-NPs for the detection of *S. aureus* 16S rRNA. HP1 was biofunctionalized with thiol groups and a fluorescent chromophore, and a thiol group was attached to the NP surface. The addition of HP2 causes the target sequence to walk along the surface of the Au-NPs, thus opening the hairpin structure of HP2 and enabling the recycling of the target sequence. They achieved a LOD of 7.73 CFU mL^−1^ with an FM of 4.36 × 10^−5^, demonstrating a novel and efficient method for the detection of *S. aureus* [116].

### 2.4. Aptamer Applications

Aptamers are short sequences of RNA or DNA (oligonucleotides), capable of folding into unique three-dimensional structures and binding to targets such as proteins, lipids, ions, small-molecular-weight metabolites, even whole cells with high specificity and affinity [117]. To produce aptamers, the SELEX (Systematic Evolution of Ligands by Exponential Enrichment) process is utilized. In this process, aptamers are generated through in vitro synthesis of combinatorial libraries with diverse sequences [53]. Through an iterative selection process, aptamers with higher affinity for the desired target are enriched and amplified, while those with lower affinity are discarded. This enables the generation of highly specific and high-affinity aptamers for various biomolecular targets [118]. The analytes of aptamer-based biosensors can vary in size and complexity as it can detect specific molecules such as proteins or more complex analytes such as whole cells. Aptamers modify their structure once reacting with a specific analyte and the conformational change can be transduced using different types of signals such as optical or electrochemical [49,119].

Some of the applications of aptamer-based sensors were developed for the detection of *S. aureus*, *E. coli* and *C. jejuni* pathogen biomarkers [53]. Another example was the results in *S. aureus* detection protocols, where they designed an ultra-sensitive magnetic fluorescence aptasensor based on fluorescence-resonance energy transfer, and the aptamers were placed on the surface of Fe_3_O_4_ and modified carbon dots (CDs). CDs were used as the fluorescence donor and Fe_3_O_4_ as the “off-on” sensor receptor. Due to the strong affinity of the aptamers to bacteria, the presence of target bacteria led to the disassembly of the Fe_3_O_4_/CDs aptasensor, resulting in the recovery of CDs fluorescence with a range of detection exhibited between 50 × 10^7^ CFU mL^−1^ and 8 CFU mL^−1^ [120].

Another example is the application of *E. coli* detection using graphene oxide (GO)-modified Au-NPs, enhanced with aptamers; an E8 aptamer was used for *E. coli* detection. The detection limit was found to be 10 cells/mL in water and coconut water-enriched samples. Furthermore, the aptamer-based nanosensor exhibited selectivity towards its target without any cross-reactivity with other bacteria. The color changes from red to blue, based on aggregation, can be easily seen by the naked eye [121]. Another protocol for *E. coli* detection in water was the nanobiosensor using QDs functionalized with aptamer II and coated with magnetic NPs. Fluorescence values were recorded for 100, 200, 300, 400, and 500 CFU, each with CdTe-MPA QDs at 100 μg mL^−1^, resulting in digital signals of 29.3 mV, 34.18 mV, 39.06 mV, 43.94 mV, and 48.82 mV, respectively, demonstrating that CdTe-MPA QDs conjugated with aptamer II were capable of selectively capturing and detecting *E. coli* [122].

Aptamers exhibit significant advantages to their application in pathogen detection, including lower molecular weight, easier and more cost-effective production methods, and good chemical stability [53]. Moreover, their ability to be generated against a wide range of targets ranging from small molecules to large proteins, and even whole live cells [123], has led to their utilization in various pathogen-detection nanobiosensor-based technologies, combined with different technologies including surface plasmon resonance (SPR), electrochemistry, piezoelectric effect, and chemiluminescence [80].

For example, SPR sensors utilize the reflection of light on a modified metal surface to detect changes using the biomarker binding in the refractive index, resulting in the precise and sensitive detection of the biomarker target [80]. In the case of electrochemical sensors, the analyte interaction is translated into an electrical signal, providing a quantitative means of detection and enabling real-time measurements [124]. Piezoelectric sensors, on the other hand, leverage the piezoelectric effect to convert the mechanical energy generated by analyte interaction into electrical energy, allowing highly sensitive and accurate detection [125]. Furthermore, chemiluminescence is another technology used in biosensors, where the analyte interaction triggers a chemical reaction that generates light. This emitted chemiluminescent light can be measured to detect and quantify the analyte, providing highly sensitive and specific detection [71]. Functionalizing nanomaterials with aptamers has allowed the combination of various signal-transduction strategies for detecting foodborne pathogens.

**Figure 3:**
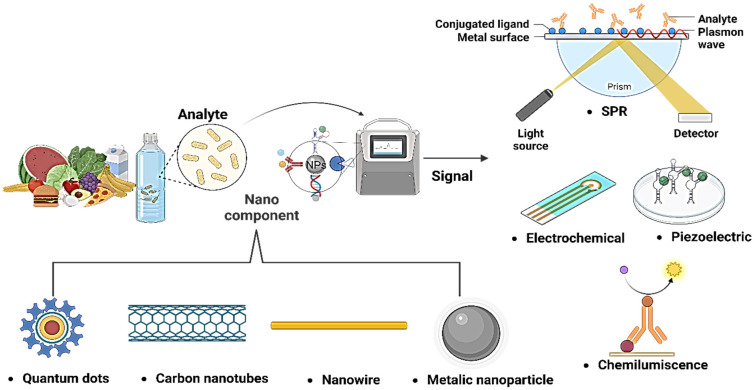
Combination of different nanomaterials and sensors for the detection of pathogens.

This detection versality provides the capability to utilize different approaches according to specification of each application, enhancing the sensitivity and selectivity of detection systems. Thus, aptamers are a powerful and promising tool in the fight against food contamination and the protection of public health [126]. These technologies enable the detection and quantification of substances in biological samples, providing versatile and efficient options for applications in fields such as medical diagnosis, food safety, and environmental monitoring (Figure 3).

### 2.5. Molecularly Imprinted Polymers (MIPs)

MIPs are defined as a group of biomimic compounds that replicate the natural interactions between a biorecognition section (antibody, antigen, or enzyme) and a biomarker; these compounds have a “lock and key” bonding mechanism to interact with the molecule of interest [127]. MIP development methods can be divided into the following: (a) covalent, (b) semi-covalent, and (c) non-covalent methods. These are in concordance with the site of action-binding modes; in general, the methods are as follows: bulk, suspension, emulsion, precipitation, multi-step swelling, and surface imprinting electrochemical polymerization [128].

In recent years the application of MIPs-based techniques has been applied to detect pathogens related to foodborne illnesses. In fact, in the case of bacteria detection, MIPs can be divided depending on the detection target (whole cells or cell membrane subunits), and subdivided in to microcontact/stamp imprinting (with a LOD of 70 CFU mL^−1^ of *E. coli*) [129], drop coating (with a LOD of 1.6 × 10^8^ cells mL^−1^ of *E. coli* strain) [130], Pickering emulsion interfacial imprinting (with a LOD of 1 × 10^3^ CFU mL^−1^ of *L. monocytogenes* strain) [131], and electropolymerization (with a LOD of 4 CFU mL^−1^ of *S. aureus* strain), among other methods that have been proved, through their LODs, to have a high detection sensitivity for foodborne pathogen [129].

## 3. Optical and Electrochemical Nanobiosensors

**Figure 4:**
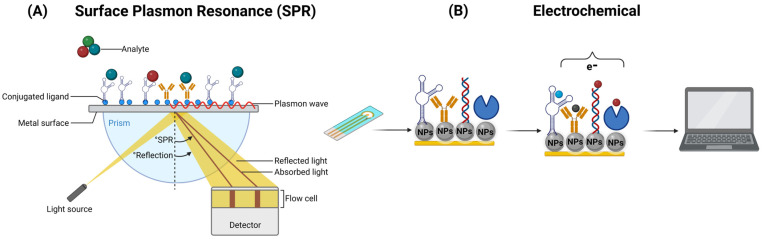
Operation of an optical SPR (**A**) and an electrochemical (**B**) biosensor, respectively.

Nanobiosensors play a crucial role in the detection of biomolecules in food and water through two distinct phenomena. Optical nanobiosensors are based on the phenomenon of the interaction of optical nanostructures with light. When specific biomolecules bind to the analyte in the sample, they trigger changes in the optical properties of light, such as absorbance or fluorescence [132]. These changes are detected and quantified to determine the presence and concentration of the analyte. Optical nanobiosensors offer high sensitivity and selectivity by harnessing this phenomenon of light–matter interaction, ensuring precise detection in food and water quality-control applications [69,70]. The Surface Plasmon Resonance (SPR) phenomenon has fundamental applications in the detection of pathogens in food and water. This sensitive and specific optical technique is used to identify the presence of pathogens (bacteria and viruses) in food and water samples. Changes in the SPR resonance angle reveal the interaction between surface biomolecules and pathogens, allowing for rapid and accurate detection of potential microbiological contaminants in these critical products for public health [132,133] (Figure 4A). On the other hand, electrochemical nanobiosensors rely on the phenomenon of electrochemical reactions on nanostructured electrodes. These electrodes provide a large surface area, enabling profoundly sensitive and selective detection. When specific biomolecules in the sample interact with the analyte, changes in electrical current or electrical potential occur on the electrode’s surface, modifying the electrical properties of the solution and generating a detectable signal (Figure 4B) [68].

Both types of nanobiosensors can be miniaturized for portable applications and are essential for ensuring the safety and quality of food and water, with the choice of nanobiosensor type depending on the specific properties of the analyte and the goals of the application at hand. Leticia Tessaro et al. (2022) [133] delve into the utilization of AuNPs in an SPR nanobiosensor designed for SARS-CoV-2 detection. While this method boasts sensitivity and precision on par with traditional RT-qPCR techniques, the cost associated with AuNPs may hinder widespread adoption. Nevertheless, it has achieved a detection time of 100 min and an LOD of 1 ng mL^−1^ (equivalent to 2.7 × 10^3^ copies per µL), establishing itself as capable of detecting the virus on food surfaces, thus emphasizing its potential in safeguarding food during pandemics.

In a similar applications, Jiayun Hu et al. (2018) [134] demonstrated the exceptional plasmonic properties of AuNPs for LSPR-based detection, offering high sensitivity with an LOD of 10 CFU mL^−1^ in *Pseudomonas aeruginosa* detection. The cost aspect remains a concern for large-scale applications. Moreover, the versatile LSPR whole-cell detection scheme demonstrated they can be extended to other microorganisms, including various bacteria and viruses, through the use of different affinity agents. This robust LSPR detection platform holds promise for clinical applications, owing to its rapid detection capability of approximately 3 h, making it suitable for point-of-care and field-based applications. Ajinkya Hariram Dabhade et al. (2023) [135] introduce AgNPs in an electrochemical biosensor for *E. coli* detection, showcasing cost-effectiveness and simplicity. This sensor demonstrates good selectivity and stability, with an LOD of 150 CFU mL^−1^. The ease of synthesis and their reproducibility make AgNPs a practical choice for on-site, real-time detection applications. Faezeh Shahdost-Fard et al., 2023 [136], introduce a unique nanocomposite-comprising sponge, copper tungsten oxide hydroxide, and AgNPs. While the synthesis process may be complex, this nanomaterial exhibits impressive performance in *S. aureus* detection. The nanocomposite-based electrochemical aptasensor offers a low LOD of 1 CFU mL^−1^ and high specificity. Its applicability in clinical samples underscores its potential for addressing nosocomial infections. The work carried out by Singh et al., 2018 [137], employed gold nanoparticles (GNPs) in a rapid-pathogen-detection assay, capitalizing on colistin’s interaction with lipopolysaccharides and the optical properties of the nanomaterial. This cost-effective approach eliminates tedious sample-preparation steps, offering a rapid, sensitive, visual detection method within 5 min. While GNPs are versatile, their sensitivity for pathogen detection at low concentrations may require further optimization for specific applications, as it exhibited a LOD of 10 cells mL^−1^ in tap water and 100 cells mL^−1^ in lake water samples. Zeynep Altintas et al., 2018 [138], present a fully automated microfluidic electrochemical biosensor designed for real-time bacteria detection. It employs immunoassays, including nanomaterial-amplified assays, to quantify *E. coli* concentrations. The sensor achieved a LOD of 1.99 × 10^4^ CFU mL^−1^ using nanomaterial amplification.

Srijit Nair et al., 2018 [139], introduce a novel approach for detecting uropathogenic *E. coli* (UPEC) using crossed surface-relief gratings (CSRGs) as nanometallic sensors. This optical-sensing platform leverages SPR-based light energy exchange for real-time, selective, and label-free UPEC detection. The LOD is reported at 10^5^ CFU mL^−1^, which is clinically relevant to urinary tract infection (UTI) diagnosis.

Olja Simoska et al., 2019 [140], focus on real-time electrochemical detection of phenazine metabolites produced by *P. aeruginosa*. Transparent carbon ultramicroelectrode arrays (T-CUAs) are used to monitor the concentrations of pyocyanin (PYO) and other metabolites. Although this work primarily centers on metabolite detection, it offers valuable insights into real-time monitoring. The study provides detailed information about phenazine dynamics over time.

## 4. Nanomaterials for the Detection of Pathogens in Water and Food

As is mentioned above, one of the major concerns in food and water safety is the precise detection of pathogens, this has led, in combination with novel sensor technologies, to an increasing exploration of nanomaterials in combination with highly efficient aptamers to revolutionize the pathogen detection in water and food. This fusion of nanotechnology and aptamers opens new possibilities for more effective control and quicker responses to potential public health risks. The following Table 1 summarizes the last five years of nanobiosensor production for the detection of viruses, bacteria, and parasites using aptamers in complex matrices.

**Figure 5:**
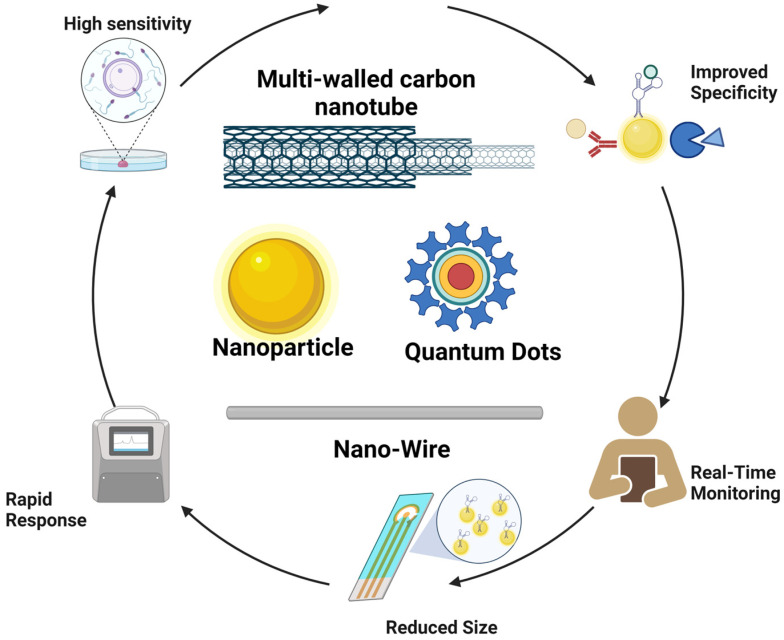
Strengths of nanobiosensors.

Nanobiosensors, due to their small size and high sensitivity, enable the real-time detection of low concentrations of biomarkers, a crucial characteristic in applications of food and water monitoring. This versatility allows them to adapt to various molecules and technologies, such as artificial intelligence incorporation. Moreover, they are more cost-effective and environmentally friendly than conventional techniques. Their miniaturization capability makes them ideal for portable devices and on-site diagnostic systems, providing quick and efficient access to quality testing and analysis in food and water. This makes them promising tools in various scientific and technological applications (Figure 5).

### 4.1. Gold Nanopartícles (Au-NPs)

Among the different types of NPs, metallic nanoparticles (MNPs) exhibit many useful characteristics such as high surface-to-volume ratio, conductivity, selectivity, and excellent optical and chemical properties, for their application in the biotechnology field [164,165]. The application can vary depending on the metal used, size, shape, surface properties, and functionalization of the MNPs [166]. On one hand, Au-NPs have been successfully used in pathogen detection because they can easily be conjugated with recognition and biorecognition elements such as aptamers, DNA, antibodies, carbohydrates, and proteins, which can enhance the reactivity and selectivity of the NPs towards specific pathogens [51,167].

In fact, Au-NPs are one of the most stable MNPs, not to mention their unique characteristics such as good chemical reactivity, conductivity, and high resistance, which have attracted attention for their use in biosensor development [168]. The surface of Au-NPs has been functionalized with various biocomponents [169]. These nanobiosensors have a very low LOD for different chemical and biological analytes, not to mention their high stability against oxidation [168]. Also, their characteristics, such as stability, conjugation, amplification properties, and their ability to serve as colorimetric biosensors [170,171,172] are especially relevant in the case of Au-NPs due to their localized surface plasmon resonance, which is a phenomenon that gives unique optical properties to MNPs, particularly Au-NPs. This is due to the interaction of electromagnetic waves with NPs of specific sizes and shapes, resulting in differential absorption of the light spectrum and different colors exhibited by the NPs [50,51]. These properties can be altered in the presence of different analytes, making Au-NPs highly suitable for biosensor development.

### 4.2. Silver Nanoparticles (Ag-NPs)

Ag-NPs stand out for their wide range of applications. These nanomaterials have been incorporated into textiles, healthcare products, consumer goods, medical devices, and biodetection applications, among others [173]. These materials are highly attractive in diagnostics field due to high conductivity, catalytic activity, and plasmonic properties presented, which may be leveraged to enhance the biosensor’s performance [174]. Sensitivity is a crucial factor for biosensors to detect low concentrations of biomarkers. Ag-NPs have been used to increase the electroactive surface area of electrodes, enhancing the electron-transfer rate and improving biosensor sensitivity [173,174]. In the incorporation of Ag-NPs in biosensor structures, Ag-NPs can amplify signals or improve the detection of nucleic acids. Their plasmonic resonance absorption band, below 500 nm, confers selective absorption in the visible and near-infrared spectrum [168]. In connection with pathogen detection, the phenomenon of surface plasmon resonance (SPR) works using the electrons on the surface of a metal, which are excited by photons of specific wavelengths and incidence angles [175,176] and applied to target detection based on the refractive index [175]. This is achieved when the biomarker is bound to a biorecognition element of the biosensor, the recognition event between the biomarker and the biorecognition element results in a change in the SPR resonance angle [31]. Conjugated polymers, such as those that include silver nanoparticles are promising materials for addressing the current and emerging issues such as pandemic monitoring [177], and pathogen detection both in food [148] and water [146].

### 4.3. Carbon-Based Nanoparticles

Similar to Au-NPs, carbon-based NPs are useful for the implementation of detection techniques for pathogen monitoring in water [119,178]. Carbon-based NPs such as carbon nanotubes, graphene, and carbon nanodots have great potential in the biosensing of pathogens because of their ability to be coated with different biomolecules for the association of molecular patterns from pathogens and to generate a signal for specific pathogens as functionalized NPs can mimic the specific surface structure of pathogens [179]. Carbon NPs have been used in the fluorescence resonance energy transfer (FRET) technique with quantum dots as donors modified with aptamers for the detection of *Vibrio parahaemolyticus* and *S. Typhimurium* in the range of 25 to 35 CFU mL^−1^ and up to between 50 and 10^6^ CFU mL^−1^, respectively [180]. Also, these NPs can be used in combination with aptamers to amplify the sensitivity and specificity of the device.

### 4.4. Magnetic Nanomaterials (MNPs)

Magnetic NPs possess their own versatility when used for biosensing pathogens, because of their specific attributes, particularly fast separation and concentration, that makes them easy tools for pathogen detection [181]. MNPs have been used for detecting pathogens using nucleic acid detection and quantification in devices for point-of-care testing in the detection of the Hepatitis B virus (LOD of 50 IU mL^−1^) and SARS-CoV-2 (500 copies mL^−1^) [182]. Magnetic NPs (MNPs) can conform to a section of the transducer part of the biosensor, or be suspended in solution in direct contact with the analyte of interest [183]. When the MNPs are in contact with the sample, they bind to the target molecule through the interaction of the label in the NPs (a functional group) and a protein; once the complex of MNPs and target is formed, an external magnetic field attracts it to the active-detection surface, and after a wash of the unbinding molecules, targets are detected [184].

When talking about magnetic NPs in biosensing, it is important to mention the magnetic relaxation switching mechanism (MRS). This phenomenon describes the incidence when cross-linking occurs between the MNPs in the binding and recognition of targets. When these MNPs clusters are formed, a change in the transverse relaxation of the sample is reflected as motional averaging or static dephasing according to the MNPs cluster size and this change can be monitored using nuclear magnetic resonance [185].

### 4.5. Silica Nanoparticles (Si-NPs)

Si-NPs have applications in the biomedical field [186], and they present good optical properties and good biocompatibility [187]. NPs are mesoporous, so in combination with other metals, have attractive and profitable characteristics for biosensing purposes [188]. Their uniformity and easily changed pore size among the gating mechanism makes it very useful in biosensing for drug delivery, for example [189]. Another important characteristic of Si-NPs is that they are considered as a GRAS (generally recognized as safe) material by the FDA [190,191]. The mesoporous nature of the Si-NPs is characteristic of a large interest, this feature can be employed to separate bacteria from complex samples even preserving its viability, and colloidal stabilization of magnetic NPs for the same purpose. Also, the silanol functional groups from SiNPs make possible the use and design of various bio-recognition systems that help to increase their sensibility and selectivity while reducing the detection time of different pathogens [190].

### 4.6. Quantum Dots (QD)

Quantum dots (QD) are colloidal nanocrystalline semiconductors that possess properties such as a quantum confinement effect, allowing them to emit and absorb light at specific wavelengths [191]. Because of this, QDs exhibit excellent optical properties, including a broad absorption spectrum, a narrow emission spectrum, and tunable luminescence, which show great prospects in biodetection [192]. QD-based biosensors include but may not be limited to fluorescence, bioluminescent, chemiluminescent, and photoelectrochemical approaches [193]. Some of the characteristics that make the use of quantum dots attractive for biosensing applications are that they possess high-quantum yield, better photobleaching resistance, wide absorption spectra, a narrow emission spectrum and their specificity with biologic targets in comparison with common fluorophores and dyes [194]. Also, it is very remarkable that its surface is easily functionalized with biologic components in order to integrate QD probes [193]. In the field of nanomaterials, the use of combinations of magnetic compounds displays attractive characteristics for current applications; these nanocomposites, besides maintaining complementary magnetic behavior, add functional proprieties to the final product [159].

As presented above, numerous studies focus their determinations on *S. Typhimurium* mainly because it is the most common pathogen related to food poisoning in Western countries causing gastroenteritis [195]. If well-used as the model or the target of the experimentations, the modifications in for example primers’ design or binding proteins may allow the replication of studies carried with this strain to any other food pathogens [161,162,196].

## 5. Prospects and Limitations to Detecting Pathogens with DNA Using Nanobiosensors

The importance of exceptionally responsive devices is essential to advancing biosensors applied in pathogen detection. Insufficient sensitivity and affinity towards biomarkers can significantly impact the performance of the device and prevent the pathogen detection. In fact, some of the biomarkers are at an ultra-low concentration in the samples (pM), and for this reason, it is necessary that the device is available to detect these ultra-low concentrations [197]. These concentrations of pathogen biomarkers in the samples present a limitation to the performance of the detection methods, this is related to the source and nature of the target biomarker itself [198]. However, through using a genetic and whole-cell-based biomarker target, and adequate sensor technologies, some of the biosensors have LOD of three genetic copies per sample [199], 1 CFU mL^−1^ [200] or have even reached an LOD of 3 × 10^6^ gene copies per sample [9], or 5 × 10^4^ CFU mL^−1^ biomarker concentration in the sample, this is considered an ultrasensitive detection range [201].

As is mentioned above, the choice of signal recognition technology can determine the sensitivity level required to identify biochemical, genetic, or whole cell biomarker concentration in the sample [132,202] and affect the performance of the device. In fact, the detection’s ultra-low concentration of pathogen-disease biomarkers concentration in samples is a mandatory requirement for early detection in clinical diagnoses [203]. Some of the disadvantages of biosensor-based pathogen detection are as follows: The factor to be determined is the target molecule to be sensed where sensitivity and specificity are compromised by the biomarker choice [109]. The use of genetic markers leads to a more sensitive device. However, it implicates complex systems and laborious sample/re-agent handling procedures [204].

On the other hand, signal emission technology is another important factor in pathogen detection. Colorimetric-based biosensors have several factors that may alter their detection capability, such has colorimetric substrate, incubation time, and even the temperature at which the signal is measured [205]. Particularly in DNA-based biosensors, other factors are lack of ability to form a complex, complication in large-scale patterns, reaction induction by mistake, and high sensitivity to enzymatic degradation and oxidation [109]. However, despite the disadvantages, there are several applied technologies in the biorecognition element of the biosensor device. For example, gene-sequence biomarkers such as CRISPR/Cas9-based technology, where the lowest LOD was three genetic copies per sample, reaching up to 3 × 10^6^ gene copies per sample [9]. In this study, the detection signal was the dose-response intensity. CRISPR/Cas12 based lateral flow, where the lowest LOD was four gene copies in the sample [199]. Other limitations to consider in the application of this device is the ability of the biosensor to discriminate between live/dead cells (LOD 1 CFU mL^−1^); the use of functionalized NPs with bacteriophages as a biorecognition agent is a solution applied for successful discrimination between live/dead [200].

One of the most astonishing advancements in the field of biosensors is the implementation of artificial intelligence and other informatic technologies in pathogen detection. The combination of artificial intelligence and biosensors has created an interdisciplinary concept of AI biosensors. The basic architecture of AI biosensors consists of three main elements: information gathering, signal conversion, and AI data processing [206]. A nanobiosensor with AI offers advantages in terms of sensitivity, speed, and analytical capability compared to conventional biosensors. This makes it suitable for application where highly precise and rapid detection of biomarkers is required, such as advanced medical diagnostics, environmental monitoring of molecular-level contaminants, and nanoscale quality control in the food industry [206,207]. The study conducted by Taniguchi et al. (2021) [208] revealed that by utilizing nanopores in conjunction with artificial intelligence, the identification of similarly sized coronaviruses is achievable. This capability has the potential to differentiate between various types of coronaviruses, such as HCoV-229E, SARS-CoV, MERS-CoV, and SARS-CoV-2. Furthermore, this technique demonstrated its effectiveness by successfully detecting SARS-CoV-2 in saliva samples. In summary, solid-state virus-immunodetection techniques hold a promising outlook for the development of versatile, adaptable, and cost-effective diagnostic tools in the future [202].

## 6. Conclusions

Over the years, significant advancements have been made in the field of biosensors, particularly in areas related to food safety and the monitoring of pathogenic microorganisms associated with food and waterborne illnesses. Despite these achievements, progress in technologies for the development of pathogen-detecting biosensors remains a highly promising area of study. This is due to the presence of various nanomaterials (MNPs, QD, carbon nanotubes, among others) with specific properties that enable the identification of specific pathogens and enhance the performance of the devices.

The nanomaterials used in biosensors offer unique advantages for pathogen detection. Thanks to their small size and large specific surface area, they facilitate more effective interaction with pathogen biomarkers, enhancing the sensitivity and selectivity of biosensors. Furthermore, their capacity to be functionalized with specific molecules, such as antibodies, nucleic acids, or aptamers, provides intrinsic advantages in the selectivity and sensitivity of the devices. Particularly with the aptamers, due to their chain-like structure, they offer greater flexibility and ease of design, making them highly selective and sensitive agents for the precise detection of pathogens. In fact, aptamers, functionalized aptamers, and other genetic-based biomarker-detection technologies have promising applications for the enhanced specificity and selectivity that has been proved in pathogen monitoring. Also, this technology compared with antibody techniques has several advantages such as improved specificity and the ability to detect genetic material, rather than proteinic, of structural biomarkers. In addition, pathogen detection through biosensors has a substantial impact on public health. The presented revision shows how nanobiosensors’ technology contributes to the precise and rapid identification of pathogenic agents in food and water, and if applied correctively, can prevent disease outbreaks and prompt appropriate measures to ensure consumer safety.

In summary, the nanomaterials employed in biosensors present diverse advantages for pathogen detection. Their reduced size, high-specific surface area, functionalization capacity, and signal amplification properties contribute to the sensitivity, selectivity, and precision of biosensors. Future possible applications of DNA-based technologies in combination with nanoparticles’ formulation, particularity the application of aptamer technologies and nanoparticles with DNA probes, will have more sensitive and specific detection techniques. In addition, this type of biosensor has the lowest capacity for detection limits by using a genetic fingerprint to discriminate between pathogens, and in the future between nonpathogenic strains, and strains of concern. These qualities, combined with the potential to use detection techniques such as fluorescence, and the application of digital technologies such as IA models, has huge potential to improve the detection capacity of the monitoring methods, creating nanomaterial-based and aptamer-based biosensors, and promising tools for pathogen monitoring and detection, enhancing safety in the food industry and public health overall.

## Figures and Tables

**Table 1:** NPs application for detection of pathogenic bacteria in food and water matrices.

Nanomaterial	Pathogen	Matrix	LOD	Signal	Bioconjugate Material	Reference
Iron core gold NPs	* S. enteritidis *	Beverage samples	32 *Salmonella* mL^−1^	Fluorescence	Antibody	[63]
FeO-NPS and Quantum dots	*E. coli*	Water	1 × 10^2^ CFU	Fluorescence	Aptamer	[122]
NAC (N-acetylcysteine) monomer	*L. monocytogenes*	Milk and pork meat	1 × 10^3^ CFU mL^−1^	Fluorescence	MPIs	[131]
Au-N triangles	* P. aeruginosa *	Water	1 cell	LSPR	Aptamer	[134]
Ag-NPs	* E. coli *	Water	150 CFU mL^−1^	Electrochemical	Aptamer	[135]
AgNPs	* S. aureus *	Bacterial suspension and human serum	1.0 CFU mL^−1^	Electrochemical	Aptamer	[136]
Au-NPs	*S. aureus*	Tap water	10^1^ to 10^4^ CFU mL^−1^	Fluorescence	Aptamer	[141]
AuNPs	*S. aureus*	Luria-Bertani media	1.5 × 10^7^ cells mL^−1^	Colorimetric	Aptamer	[142]
AuNPs	*Ochratoxin A*	Peanut, soybean, and corn	28.18 pg/mL	Colorimetric	Aptamer	[143]
AuNPs	* E. coli *	Flour	2.5 ng µL^−1^	Colorimetric	Probe	[144]
Graphene oxide coated AuNPs	*E. coli* *S. Typhimurium*	Bacterial suspension	1 × 10^3^ CFU	Colorimetric	Antibody	[145]
Ag-NPs	* S. aureus *	Water	1.0 CFU mL^−1^	Electrochemical	Aptamer	[146]
Chitosan-AgNPs	* Glipopolysaccharide *	Bacterial suspension	248 CFU mL^−1^	Electrochemical	-	[147]
AgNPs	*E. coli*	Pork, cabbage and milk	2.0 CFU mL^−1^	Photoelectrochemical	Peptide Magainin	[148]
Au-NPs and oxide of graphene NPs	*E. coli*	Water	9.34 CFU mL^−1^	Electrochemical	Aptamer	[149]
Multiwalled carbon nanotubes	*E. coli*	Water	0.8 CFU mL^−1^	Electrochemical	Antibody	[150]
Graphene and carbon nanotubes	*Salmonella enteritidis*	Water	10^2^–10^8^ CFU mL^−1^	Colorimetric	Antibody	[151]
Quantum dots	*S. aureus*, *S. Typhimurium*	Water	16–28 CFU mL^−1^	Colorimetric	Aptamers	[152]
SiNPs	*E. coli*	Bacterial suspension	10^3^ CFU mL^−1^	Electrochemical	Polyclonalantibodies	[153]
SiNPs	*E. coli*	Bacterial suspension	8 CFU mL^−1^	Fluorescence	Rhodamine B	[154]
SiNPs	AFB1 from filamentous fungi	Peanut, maize, and badam	0.214 pg mL^−1^	Fluorescence	Aptamer	[155]
MNPs	* S. aureus *	Milk, Romaine lettuce, ham, and sausage	2.5 ng µL^−1^	Colorimetric	Probes	[156]
Iron oxide MNPs assisted AuNPs	*B. cereus* and *Shigella flexneri*	Inoculated media	12 cells mL^−1^ and 3 cells mL^−1^	Electrochemical	Vancomycin	[157]
Magnetic NPs	*S. Typhimurium*	Food	53 UFC/mL	Fluorescence	Oligonucleotides	[158]
Iron oxide encapsulated quantum dots	Hepatitis E virus Norovirus	Clinical samples	56 RNA copies mL^−1^ 69 RNA copies mL^−1^	Fluorescence Electrochemical	Antibody	[159]
QDs	*S. Typhimurium*	Chicken meats	43 CFU mL^−1^	Fluorescence	Antibody	[160]
QDs	*S. Typhimurium* and *V. parahaemolyticus*	Aquatic samples	10 CFU mL^−1^ 10^2^ CFU mL^−1^	Fluorescence	Aptamer	[161]
QDs nanobeads	*S. Typhimurium*	Potable water, orange juice, lettuce, and chicken	10^−1^ CFU mL^−1^	Fluorescence	Antibody	[162]
TAA *, TBA **, TMA *** and TE ****	*S. aureus*	Lettuce/Shrimp	4 CFU mL^−1^	Electrochemical/Fluorescence	MPIs	[163]

Abbreviations are referred to the following compounds: * 3-thiopheneacetic acid, ** 3-thiopheneboronic acid, *** 3-thiophenemethylamine, **** 3-thiopheneethanol.

## Data Availability

Not applicable.
